# The Crosstalk Between Hippo-YAP Pathway and Innate Immunity

**DOI:** 10.3389/fimmu.2020.00323

**Published:** 2020-02-27

**Authors:** Shuai Wang, Lili Zhou, Li Ling, Xuli Meng, Feng Chu, Suping Zhang, Fangfang Zhou

**Affiliations:** ^1^Institutes of Biology and Medical Sciences, Soochow University, Suzhou, China; ^2^Department of Breast Surgery, Zhejiang Provincial People’s Hospital, Hangzhou, China; ^3^Guangdong Key Laboratory for Genome Stability and Human Disease Prevention, Department of Pharmacology, Base for International Science and Technology Cooperation: Carson Cancer Stem Cell Vaccines R&D Center, International Cancer Center, Shenzhen University Health Science Center, Shenzhen, China

**Keywords:** Hippo-YAP pathway, innate immunity, type I interferon, NF-kB signaling, tumor development

## Abstract

Recognition of pathogen-associated molecular patterns (PAMPs) triggers expression of antiviral interferons and proinflammatory cytokines, which functions as the frontier of host defense against microbial pathogen invasion. Hippo-YAP pathway regulates cell proliferation, survival, differentiation and is involved in diverse life processes, including tissue homeostasis and tumor suppression. Emerging discoveries elucidated that the components of Hippo-YAP pathway, such as MST1/2, NDR1/2, and YAP/TAZ played crucial regulatory roles in innate immunity. Meanwhile the innate immune signaling also exhibited regulatory effect on Hippo-YAP pathway. As for the importance of these two pathways, it would be interesting to figure out the deeper biological implications of their interplays. This review focuses on the regulation between Hippo-YAP pathway and innate immune signaling. We also propose the possible contribution of these interplays to tumor development.

## Introduction

Hippo-YAP pathway spurs intense research interest owing to its remarkable biological functions in tissue homeostasis, development, and cancer. The Hippo-YAP pathway relays diversified extracellular and intracellular signals, including cell density, cell polarity, mechanical cues, ligands of G-protein-coupled receptors, cellular energy status, and orchestrates cell proliferation, survival, apoptosis, and differentiation and stemness. Dysregulation of Hippo pathway has important implications in tumorigenesis, progression, and metastasis (1–8).

**FIGURE 1:**
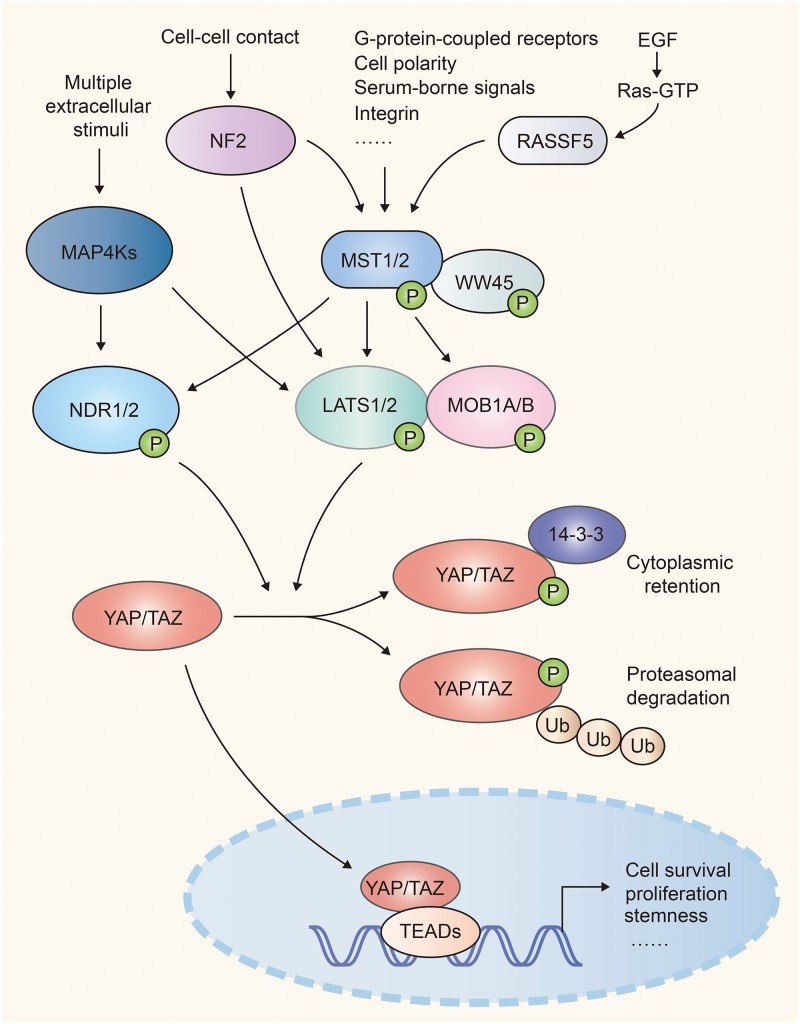
A simple scheme of the Hippo pathway in mammalian cells. NF2 and RASSF family proteins (e.g. RASSF5) function upstream and facilitate the activation of Mst1/2. MST1/2 and MAP4Ks relay diversified extracellular and intracellular signals, and activate LATS1/2 and NDR1/2, which cause inhibitory phosphorylation of YAP/TAZ. When Hippo signaling is attenuated, activated YAP/TAZ translocate into nuclei and recruit TEAD transcription factors to regulate cell proliferation, survival, and stemness.

Hippo-YAP pathway was originally identified in *Drosophila* and highly conserves in mammals. Upon activation of this pathway, the MST1/2 (mammalian Ste20-like kinases 1/2, orthologs of Hippo in *Drosophila*) and its adaptor WW45 (Sav family WW domain containing adaptor 45, also called Sav, ortholog of Salvador) phosphorylate and activate the downstream kinase LATS1/2 (large tumor suppressor 1/2, orthologs of Warts in *Drosophila*). Then LATS1/2 and its adaptor protein MOB1A/B (Mps one binder 1A and B, orthologs of Mats) phosphorylate the transcriptional co-activator YAP (Yes-associated protein, Yorkie ortholog) and TAZ (transcriptional co-activator with PDZ binding motif), which results in their cytoplasmic retention via interaction with 14-3-3 or poly-ubiquitination and degradation in the proteasome. Once Hippo signaling is off, inactivated MST1/2 and LATS1/2 release YAP and TAZ from inhibitory phosphorylation, leading to their accumulation in the nucleus. YAP and TAZ then bind with transcription factors TEAD1-4 (TEA domain, Scalloped orthologs) to induce gene transcription that contribute to cell proliferation and survival (1, 2) (Figure 1).

MAP4Ks (mitogen-activated protein kinase kinase kinase kinase, orthologs of Happyhour in *Drosophila*), NDR1/2 (nuclear Dbf2-related 1/2), NF2 (Neurofibromatosis type II, also known as Merlin), and RASSFs (Ras association domain family member) were identified to be the key components of Hippo-YAP signaling. MAP4Ks function in parallel to MST1/2 in Hippo cascade, phosphorylate and activate LATS1/2 (9, 10). The kinases NDR1/2, act as an alternative YAP kinases similar to LATS1/2. NDR1/2 can be phosphorylated and activated by MST1/2 (11, 12) and phosphorylate YAP and inhibit its activity (13, 14). NF2 functions upstream of the Hippo cascades and facilitates LATS1/2 phosphorylation by MST1/2 (15, 16). RASSF family proteins, such as RASSF5, serve as the upstream regulator of MST1/2. RASSF5 induces the homodimerization, trans-autophosphorylation and activation of MST1/2 via the RASSF5-MST1/2 complex (17) (Figure 1).

Innate immunity provides the initial detection and rapid defense against invading pathogens, contributing to homeostasis and activation of the adaptive immune response. The recognition of pathogen by the innate immune system depends on germline-encoded pattern-recognition receptors (PRRs), including toll-like receptors (TLRs), retinoic-acid-inducible gene I (RIG-I)-like receptors (RLRs), cytosolic DNA sensors, C-type lectin receptors (CLRs), and NOD-like receptors (NLRs) (18–20). TLR3/7/8/9 senses viral RNA and DNA in the endosome (21). Among them, TLR3 senses viral double-stranded RNA (22); TLR7 and TLR8 prefer viral single-stranded RNA (23, 24) and TLR9 prefers unmethylated CpG DNA from bacteria and viruses (25). While TLR1/2/4/5/6/10 locate on cell surface and mainly recognize microbial membrane or cell wall components (21), TLR2 and TLR1/6 cooperatively sense lipoproteins/lipopeptides and peptidoglycans from bacteria, fungi and viruses (26, 27). In addition, TLR4 specifically senses lipopolysaccharide (LPS) (28) and TLR5 recognizes bacterial flagellin (29). The RLR family receptor RIG-I and melanoma differentiation-associated gene 5 (MDA5) are responsible for the recognition of viral RNA in cytoplasm. RIG-I recognizes short dsRNA and 5’ppp-RNA, while MDA5 prefers long dsRNA (30). A number of DNA sensors, including nucleotidyltransferase cGAS (cyclic GMP-AMP synthase) (31, 32), interferon-inducible protein 16 (IFI16) (33), RNA Polymerase III (Pol III) (34), DNA-dependent activator of IFN-regulatory factors (DAI) (35), and DEAD box protein 41 (DDX41) (36) recognize the viral DNA and activate the innate antiviral immunity.

The recognition of pathogens triggers the recruitment of adaptors to PRRs, including mitochondrial antiviral signaling protein (MAVS) (37), stimulator of interferon genes (STING) (38), Toll/IL-1 receptor domain-containing adaptor-inducing IFN-β (TRIF) (39), myeloid differentiation factor 88 (MyD88) (40), and initiates a series of signaling cascades which converge at several protein kinases, such as TANK-binding kinase 1 (TBK1), IκB kinase ε (IKKε) (41), IL-1R-associated kinase 1/2/4 (IRAK1/2/4) (42), and the IKK complexes composed of IKKα, IKKβ and NEMO (NF-κB essential modifier, also known as IKKγ) (43). These kinases further phosphorylate and activate the transcription factor like interferon regulatory factor 3/7 (IRF3/7), Nuclear Factor-κB (NF-κB), which induce the synthesis and release of diverse IFNs, inflammatory cytokines, and chemokines. IFNs subsequently activate downstream signaling pathways that induce a diverse set of interferon-stimulated genes (ISGs) and protect host cells from the invading virus. The activation of NF-κB induces the expression of proinflammatory cytokines and chemokines, which elicits inflammatory response and recruits immune cells to the site of infection (21, 44, 45). Furthermore, detection of exogenous pathogens induces the generation of reactive oxygen species (ROS), which is required for the elimination of bacterial infection. ROS-mediated host defense not only exhibits a direct microbicidal activity, but also plays regulatory roles in the activation and phagocytosis of immune cells to eliminate invading pathogens (46).

Emerging evidences have suggested that innate immune response was extensively regulated by multiple core components of Hippo-YAP cascade while the activation of innate immune signaling conversely led to substantial modulations of Hippo-YAP pathway. Several clinical cases reported that a number of immunodeficiency symptoms, including recurrent bacterial and viral infections, were associated with the deficiency of Hippo signaling, implying its critical role in immune modulation (47, 48). Yet, the overall picture of the reciprocal regulation of the two pathways remains to be fully clarified. This article reviews the relevant investigations of their reciprocal actions and discusses the possible contribution to tumor progression.

## Hippo-YAP and IFN-I Signaling

Production of type I interferon (IFN-I, including IFN-α and IFN-β) is a representative hallmark of host’s defense to viral infection. IFN-I consequently drives the production of a diverse set of ISGs and protects host cells against the invading virus.

**FIGURE 2:**
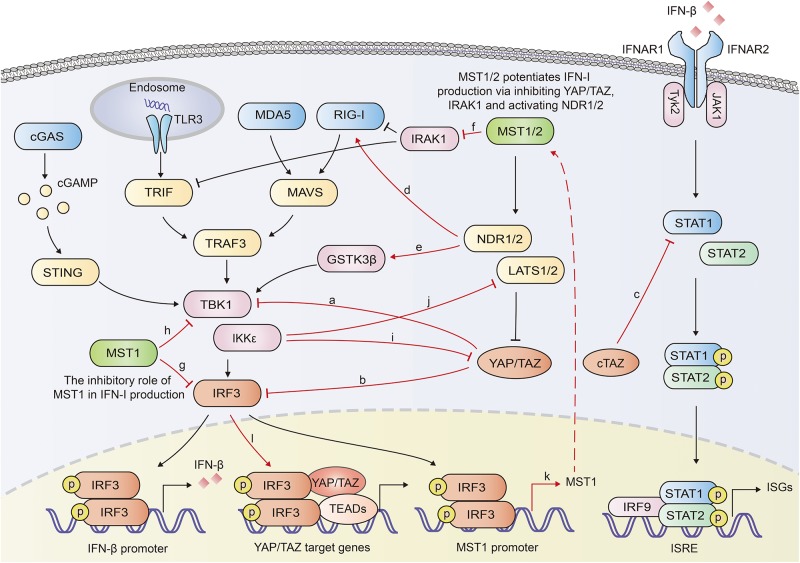
Overview of reciprocal augment of Hippo-YAP signaling and innate antiviral response. YAP/TAZ antagonizes the innate antiviral response by inhibiting the activation of TBK1 **(a)** or IRF3 **(b)**. cTAZ inhibits STAT1 activation and ISGs production **(c)**. NDR2 promotes the K63-linked polyubiquitination of RIG-I, potentiating the IFN-β production **(d)**. NDR1 facilitates TBK1 activation by mediating the activation of GSK3β **(e)**. MST1 mediates the degradation of IRAK1, a negative regulator for IFN-β production **(f)**. MST1 inhibits IRF3 **(g)** and TBK1 **(h)**, repressing IFN-β production. IKKε mediates the degradation of YAP **(i)** and LATS1/2 **(j)**. Activated IRF3 induces the transcription of MST1 and the activation of Hippo pathway **(k)**. Activated IRF3 interacts with YAP-TEAD complex and promote the activation of YAP (**l**). In general, Hippo-YAP signaling and innate antiviral signaling exhibit reciprocal activation. The interplays between Hippo-YAP and innate antiviral signaling are highlighted with red color.

YAP/TAZ was documented to inhibit the innate antiviral defense by targeting TBK1. YAP/TAZ prevented the K63-linked ubiquitination of TBK1 and disrupted its interaction with MAVS, STING and IRF3, diminishing the virus-induced IFN-I and ISGs production (49). Ectopically expressed human YAP mutant lacking transcription activity (YAP 6SA) in zebrafish embryos resulted in compromised antiviral responses, enhanced mortality and susceptibility to viral infection. LATS1/2 kinases-mediated inactivation of YAP/TAZ relieved the TBK1 suppression and enhanced anti-viral responses (49). Our previous work also identified YAP as a negative regulator of innate antiviral response (50). We found YAP interacted with IRF3, a critical transcription factor in innate immunity, and prevented its dimerization and nuclear translocation, which further reduced the production of IFN-β and ISGs in response to virus infection. This inhibition of IRF3 by YAP was independent on its transcriptional regulation function, since YAP4, an isoform lacking the amino-terminal TEAD-interacting domain, sequestered IRF3 more efficiently in the cytoplasm. Genetic deletion of YAP in mice enhanced innate antiviral response and reduced the susceptibility to viral infection (50). Similar to the shorter YAP isoform YAP4, an alternatively transcribed TAZ variant cTAZ was also reported to shut down the innate antiviral immunity. cTAZ, lacking TEAD-binding domain (TBD) and WW domain, physically interacted with STAT1, and suppressed the dimerization and nuclear translocation of STAT1/2, inhibiting the ISGs expression and antiviral response (51) (Figure 2).

Recently, NDR2 was identified as a crucial positive regulator of the RIG-I-mediated antiviral immune response. Overexpression of NDR2 enhanced the RNA virus-induced production of type I IFN and proinflammatory cytokines. Myeloid deletion of NDR2 in mice impaired antiviral immune response and increased virus load. Mechanistically, NDR2 directly interacted with RIG-I and TRIM25, thus facilitating the RIG-I/TRIM25 complex and enhancing the TRIM25-mediated K63-linked polyubiquitination independent of its kinase activity (52). Furthermore, NDR1 was shown to increase GSK3β kinase activity by reducing its phosphorylation, while GSK3β had been proven to increase the activation of TBK1 (53, 54). Overexpressed NDR1 and GSK3β in Grass carp cells up-regulated the expression of IFN I (53) (Figure 2).

Li et al. reported that MST1 increased the TLR3/4-triggered IFN-β production in macrophages. MST1 directly bound to and phosphorylated IL-1 receptor-associated kinase 1 (IRAK1), leading to the degradation of IRAK1 (55). IRAK1 has been proven to negatively regulate MyD88-, TRIF-, and RIG-I-mediated IFN-β production (56, 57). Therefore MST1-mediated degradation of IRAK1 potentiated IRF3 activation and IFN-β production (55).

On the other hand, the innate antiviral immunity was proven to extensively regulate the Hippo-YAP pathway. In our previous paper, IKKε, activated in response to viral infection induced the degradation of YAP. Mechanistically, IKKε phosphorylated YAP at Ser403 and induced its lysosomal degradation. IKKε depletion abolished YAP S403 phosphorylation and the degradation after viral infection. Mutation of YAP S403A prevented YAP degradation induced by IKKε transfection or viral infection. Given the significant inhibitory role of YAP in innate antiviral immunity, IKKε-mediated YAP degradation relieved the repression of YAP on the innate antiviral response (50). On the contrary, another group identified IKKε to be a negative regulator of the Hippo pathway via mediating degradation of LATS1/2, suggesting a complicated role of IKKε in Hippo pathway (58) (Figure 2). cGAS-induced innate immune response was demonstrated to activate Hippo-signaling by increasing the expression of MST1 (59). Palmitic acid treatment induced the release of mitochondrial DNA which was sensed by cGAS in the cytoplasm and initiated cGAS-cGAMP-STING-TBK1-IRF3 cascade. Activated IRF3 bound to the promoter of MST1 gene and induced the transcription of MST1 and subsequent YAP/TAZ inactivation (59). Given that MST1 could be induced and activated by IRF3, it is probably that the innate immune signaling that activates IRF3 may also mediate the activation of Hippo-YAP pathway. However, IRF3 was identified to be an agonist of YAP. IRF3 interacted with YAP and TEAD4 to form a complex in nuclei, promoting nuclear translocation and activation of YAP. Knocking down or pharmacological targeting IRF3 inhibited gastric tumor growth in a YAP-dependent manner (60). Various TLR ligands, including Pam3CSK4 (an agonist of TLR1 and TLR2), poly (I:C) (an agonist of TLR3), lipoteichoic acid (an agonist of TLR2), LPS (an agonist of TLR4) have been demonstrated to induce phosphorylation of Mob1, a physiological substrate of MST1 and MST2, in BMDMs (61), implying that TLRs induce the activation of MST1/2, and even further the Hippo-YAP pathway. Moreover, lipid A (a ligand of TLR2), poly (I:C) and LPS vigorously activated MAP4K2 (also named GCK, Germinal center kinase) (62), implying that TLRs might interplay with Hippo-YAP pathway via MAPKs.

The results described above suggest a reciprocal activation of Hippo-YAP pathway and innate antiviral response (Figure 2). Beyond that, another investigation demonstrated that MST1 directly phosphorylated IRF3 at T75 and T253, which disrupted the dimerization of IRF3 and restrained RLRs and cGAS-mediated innate antiviral response. MST1 also inhibited the activation of TBK1 (63). Given that activated IRF3 induced the expression of MST1 (59), MST1-mediated inhibition might serve as a negative feedback to avoid excessive activation of innate antiviral immunity. However, Boro et al. reported that MST1/2 activated IRF3 and induced the production of CXCL1 and CXCL2 in response to *Mycobacterium tuberculosi*s (*Mtb*) infection (64). Moreover, as both MST1 and YAP/TAZ dampened the innate antiviral immunity, the effect of MST1 on host antiviral defense seems sophisticated and may rely on the context involved (Figure 2).

Taken together, in most cases, YAP/TAZ restrains innate antiviral immunity, while the core kinases of Hippo signaling, MST1/2 and NDR1/2 positively regulate the innate antiviral response. The innate antiviral immunity also activates and forms a positive loop with Hippo pathway.

## Hippo-YAP and NF-κB Signaling and Inflammation

**FIGURE 3:**
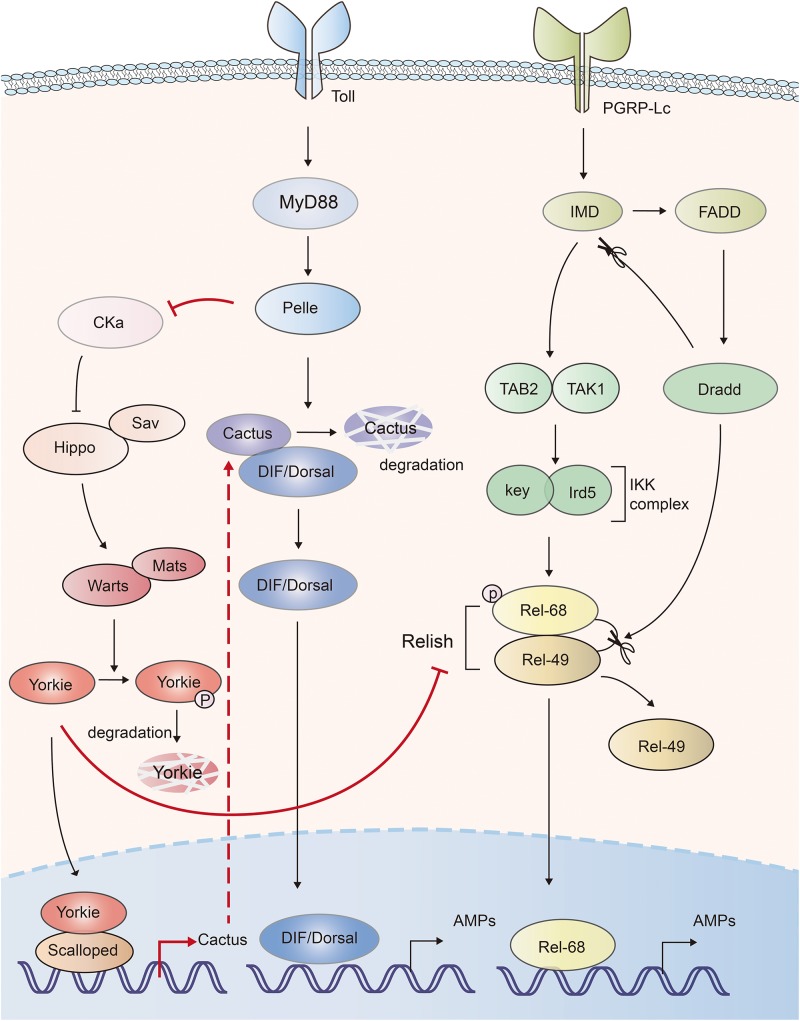
The mutual activation of Hippo-Yorkie pathway and Toll/IMD mediated anti-bacterial response in *Drosophila*. Yorkie-Scalloped complex induce the expression of Cactus, which abolishes the activity of *Drosophila* the NF-κB transcription factors dorsal/DIF and AMPs production. Yorkie also impairs the IMD pathway mediated AMPs production via suppressing the expression of Relish, the NF-κB protein in IMD pathway. Activation of Toll receptor activates Hippo via Toll-Myd88-Pelle cascade mediated degradation of Cka. The interplays between Hippo-Yorkie and Toll/IMD pathway are highlighted with red.

NF-κB pathway is essential for inflammatory and immune responses. In *Drosophila*, Toll and IMD (immune deficiency) pathways induce the expression of antimicrobial peptides (AMPs) via activation of NF-κB transcription factors, DIF (dorsal-related immunity factor), Dorsal and Relish, respectively (65). The *Drosophila* IκB factor cactus, formed complex with NF-κBs, acts as a negative regulator of innate immunity (66). Hippo-Yorkie signaling has been indicated to promote Toll-mediated anti-microbial peptide expression in response to Gram-positive bacterial and fungal infections by enhancing NF-κB signaling in *Drosophila* (67). Depletion of the Hippo, Warts or overexpression of Yorkie in the fat body increased the Gram-positive bacteria- and fungi-induced lethality to a similar level of Toll signaling-deficient *Drosophila*. Mechanistically, deficiency of Hippo signaling enhanced the activation of Yorkie, which in turn bound to its transcription factor partner Scalloped to induce the transcription of Cactus, *Drosophila* IκB factor. Cactus abolished the activity of the NF-κB transcription factors dorsal and DIF (Dorsal-related immunity factor), as well as the expression of anti-microbial peptides (67). Another similar work also indicated that Yorkie negatively regulated both Toll and IMD pathways. Yorkie overexpression or Warts knockdown in *Drosophila* significantly downregulated the synthesis of AMPs by enhancing Cactus expression and decreasing the expression of Relish, the *Drosophila* NF-κB factor in IMD pathway (68). Thus, those studies suggest that Hippo positively, and Yorkie negatively regulate the innate defense (Figure 3).

On the other hand, Hippo-Yorkie pathway is also activated by the innate immunity in *Drosophila*. Toll receptor sensed gram-positive bacteria and activated Toll-Myd88-Pelle cascade. The protein kinase Pelle in turn phosphorylated and degraded Cka subunit of STRIPAK (striatin-interacting phosphatase and kinase)-PP2A complex, an inhibitor of Hippo, leading to the activation of Hippo-YAP pathway and the augment of innate immune response (67, 69) (Figure 3). Thus, mutual activation of Hippo-Yorkie pathway and Toll/IMD-mediated anti-bacterial response might exist in *Drosophila*.

**FIGURE 4:**
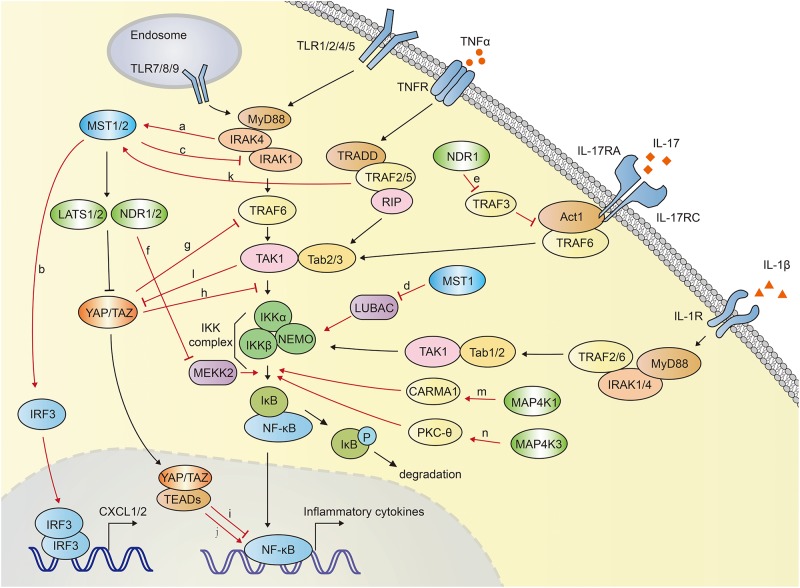
The crosstalk between Hippo-YAP pathway and NF-κB signaling. MST1/2 is activated via TLR2-MyD88-IRAK1/4 cascade during *Mtb* infection **(a)**, and subsequently induce the expression of CXCL1 and CXCL2 by activating IRF3 **(b)**. MST1 inhibits NF-κB activation via promoting IRAK1 degradation **(c)** and inhibiting LUBAC-mediated NEMO linear ubiquitination **(d)**. MST1 is activated by TRAF2 after TNFα stimulation. NDR1 promotes the IL-17-induced inflammatory response by interact with TRAF3, which disrupts the IL-17R-Act1-TRAF6 complex **(e)**. NDR1/2 inhibit inflammatory cytokine production by promoting Smurf1-mediated degradation of MEKK2 **(f)**. YAP blocks NF-κB activation via promoting the TRAF6 degradation **(g)** or disrupting the interaction between TAK1 and IKKα/β **(h)**. YAP/TAZ-TEADs complex inhibits the transcriptional activation of NF-κB targeted genes in low-density-cells **(i)**. Long-term expression of YAP/TAZ in hepatocyte potently activates the expression of inflammatory factors **(j)**. TNFα stimulation activates MST1 in a TRAF2 dependent manner **(k)**. TAK1 directly phosphorylates YAP/TAZ, resulting in their degradation independent of LATS1/2 **(l)**. MAP4K1 **(m)** and MAP4K3 **(n)** phosphorylate CARMA1 and PKC-θ, respectively, contributing to IKK and NF-κB activation. The crosstalk between Hippo-YAP and NF-κB signaling are highlighted with red color.

In mammals, Hippo-YAP pathway acts as a positive regulator of anti-bacterial and inflammatory response (Figure 4). Boro et al. identified that MST1/2 increased the *Mtb* infection-induced production of pro-inflammatory molecules and chemokine (64). TLR2 sensed *Mtb* infection and then led to the activation of MST1/2, which in turn increased the production of inflammatory chemokines CXCL1 and CXCL2 in an IRF3-dependent, but LATS1/2- independent manner. CXCL1 and CXCL2 further induced the production of anti-microbial and inflammatory molecules. Transfection of MST1/2 kinase dead mutant or knockdown of MST1/2 compromised *Mtb*-induced CXCL1 and CXCL2 production (64).

On the contrary, MST1 was reported to phosphorylate IRAK1 and induce its degradation, which further inhibited TLR4/9-NF-κB signaling and inflammatory responses in macrophages. MST1-deficient mice exhibited more severe lung damage and excessive proinflammatory cytokine secretion after challenged with LPS (55).

MST1 was also reported to attenuate NF-κB-dependent inflammatory gene expression induced by TNFα. After TNFα stimulation, MST1 was activated and recruited to TNF-RSC (TNFα receptor 1 signaling complex). This complex interacted with and phosphorylated HOIP, the catalytic component of the E3 ligase LUBAC (linear ubiquitin assembly complex). TRAF2 was required for the recruitment of MST1 to TNF-RSC. MST1 further phosphorylated HOIP, inhibited E3 ligase activity of LUBAC and LUBAC-dependent linear ubiquitination of NEMO, and finally inhibited NF-κB activation and expression of NF-κB target genes (70) (Figure 4).

MAP4K1, also named hematopoietic progenitor kinase 1 (HPK1), plays a critical role in NF-κB activation. Heterologous expression of MAP4K1 notably promoted the kinase activity of IKKα/β and NF-κB activation (71–73). MAP4K1 depletion dampened TCR (T cell receptor)-induced kinase activity of IKK and NF-κB activation in T cells. Upon TCR stimulation, MAP4K1 interacted with and phosphorylated the adaptor protein Caspase recruitment domain-containing membrane-associated guanylate kinase prote in-1 (CARMA1), which facilitated IKKβ and NF-κB activation (73, 74). MAP4K3, also named Germinal center kinase–like kinase (GLK), has been demonstrated to involved in NF-κB activation during T-cell activation (75–77). TCR signaling activated MAP4K3, which subsequently phosphorylated and activated PKC-θ (Protein kinase C-θ) and led to the activation of IKK, NF-κB in T cells. MAP4K3-deficiency abolished the PKC-θ-IKK activation and proliferation in primary T cells. Genetic ablation of MAP4K3 in mice dampened T cell-mediated immune responses (76). Similarly, in a collagen-induced arthritis model, MAP4K3-deficient mice displayed compromised inflammatory cytokine production including IL-2, IL-6, IL-17A, IL-4, and IL-1β (78). T cell-specific MAP4K3 transgenic mice spontaneously developed systemic inflammation and autoimmune diseases (79). MAP4K4, also termed hematopoietic progenitor kinase/germinal center kinase-like kinase (HGK), has been suggested to play a regulatory role in innate immunity. Knocking down of MAP4K4 with siRNA in macrophages impeded LPS-induced TNFα and IL-1β production (80).

NDR1 was suggested to be a positive regulator in IL-17 (Interleukin 17)-mediated NF-κB activation (81). IL-17 is an important inducer of tissue inflammation. NDR1 was shown to promote IL-17-induced phosphorylation of p38, ERK1/2, NF-κB and the expression of chemokines and cytokines, independent of its kinase activity. IL-17-induced inflammation was significantly reduced in NDR1-deficient mice. Mechanistically, NDR1 competed with IL-17 receptor (IL-17R) for TRAF3 binding (81), whereas TRAF3 had been shown to interact with IL-17R and disturb the formation of the IL-17R-Act1-TRAF6 complex (82). NDR1 was reported to inhibit CpG-TLR9-mediated inflammatory cytokine production in macrophages and protect host from inflammatory injury. NDR1 interacted with Smad ubiquitin regulatory factor 1 (Smurf1), an E3 ligase for mitogen-activated protein/ERK kinase kinase 2 (MEKK2), and enhanced Smurf1-mediated K48-linked ubiquitination and degradation of MEKK2 (83). MEKK2 has been identified to be indispensable for recruitment of NF-κB-IκBβ complex to IKK complex, and controlled the delayed activation of NF-κB in response to TNFα and IL-1β (84). MEKK2 is crucial for CpG-induced TNF-α and IL-6 production. Thus, NDR1-deficient mice showed robust TNF-α and IL-6 production and were more susceptible to CLP-induced sepsis and *Escherichia coli* infection (83). Similarly, NDR2 was also reported to interact with Smurf1 and promote MEKK2 degradation, impeding IL-17-induced inflammation. NDR2 deficiency increased IL-17-induced IL-6, CXCL2 and CCL20 expression (85) (Figure 4).

Lv et al. demonstrated an inhibitory role of YAP in NF-κB activation. YAP was found to interact with and promote the polyubiquitination and degradation of TRAF6, dampening TRAF6-mediated NF-κB activation. Endothelial specific YAP knockout mice exhibited enhanced the expression of NF-κB, proinflammatory cytokines IL-6, TNF-α, IL-1β, and lung inflammatory injury and mortality in response to LPS. TRAF6 depletion alleviated the robust inflammatory response in endothelial YAP-deficient mice (86). Furthermore, Deng et al. proved that YAP inhibited inflammatory responses mediated by NF-κB signaling in a murine model of experimental osteoarthritis (87). YAP overexpression disrupted the interaction between TAK1 and its downstream proteins IKKα and IKKβ, attenuating the activation of NF-κB signaling which played a major role in osteoarthritis pathogenesis via mediating the expression of proinflammatory cytokines such as TNFα (tumor necrosis factor α), IL-1β and IL-6 (88). Transgenic overexpression of YAP or genetic deletion of MST1/2 in mice alleviated osteoarthritis symptoms, whereas deletion of YAP in chondrocytes aggravated the progression of osteoarthritis (87). Additionally, in low-density-cells, YAP/TAZ translocates into the nuclei and forms YAP/TAZ-TEADs complex, functioning as a transcriptional activator. This YAP/TAZ-TEADs complex could recruit histone deacetylase 7 (HDAC7) to the promoter regions of NF-κB targeted genes, reducing the production of inflammatory cytokines (89). Nevertheless, TAZ was demonstrated to promote liver inflammation and tumor development. The expression of TAZ, but not YAP, was found to correlate with the mRNA transcription of inflammatory cytokines CCL2 and CXCL1 in human liver tumors. Expression of TAZ in mouse livers increased the secretion of proinflammatory cytokines, recruitment of myeloid cells, and mice mortality in a TEADs dependent manner (90). Mooring et al. found that the long-term expression of YAP/TAZ in hepatocyte positively correlated to the degree of liver inflammation. Hepatocyte-specific expression of a constitutive active form of YAP in mice for a week potently activated the expression of inflammatory factors including TNFα, IL-1β. In contrast, hepatocyte-specific YAP or YAP/TAZ knockouts exhibited less inflammation (91) (Figure 4).

The researches presented above suggested the sophisticated roles of Hippo-YAP pathway in the modulation of NF-κB signaling. Hippo-YAP pathway might be either positive or negative regulator of NF-κB signaling. In many cases, Hippo-YAP signaling was activated by NF-κB signaling and inflammatory responses. MST1/2 of Hippo-YAP signaling was shown to be activated via TLR2-MyD88-IRAK1/4 axis during *Mtb* infection. *Mtb* infection was recognized by TLR2, which initiated the signal transmission via TLR2-MyD88-IRAK1/4 axis. Subsequently IRAK1 and IRAK4 directly interacted with and activated MST1/2. Inhibition of IRAK1 and IRAK4 compromised *Mtb*-induced MST1/2 activation (64). Lee et al. documented that TNFα stimulation mediated the activation of MST1 (70). After the stimulation with TNFα, TRAF2 recruited MST1 to TNF-RSC, and subsequently mediated the homo-dimerization and activation of MST1. TRAF2 deficiency abolished TNFα-induced homo-dimerization and activation of MST1 (70, 92). Similarly, MST1 and LATS1 were reported to be strongly activated by inflammatory cytokines, including TNFα and IL-1β, which further promoted YAP/TAZ degradation and attenuated the expression of YAP/TAZ-targeted genes (87). Interestingly, TAK1 involved in LATS1/2-independent YAP/TAZ degradation. TAK1 directly associated with and phosphorylated YAP/TAZ, resulting in the proteasomal degradation of YAP/TAZ. TAK1 deletion significantly dampened TNFα-induced degradation of YAP/TAZ (87) (Figure 4). Furthermore, TNF-α stimulation has been demonstrated to potently activate several MAP4Ks family members, including MAP4K2, MAP4K3 (62, 93), and MAP4K5 (also named KHS, Kinase homologous to SPS1/STE20) (94, 95), suggesting the inflammatory response might activate Hippo pathway via MAP4Ks.

## Hippo Signaling and Reactive Oxygen Species

**FIGURE 5:**
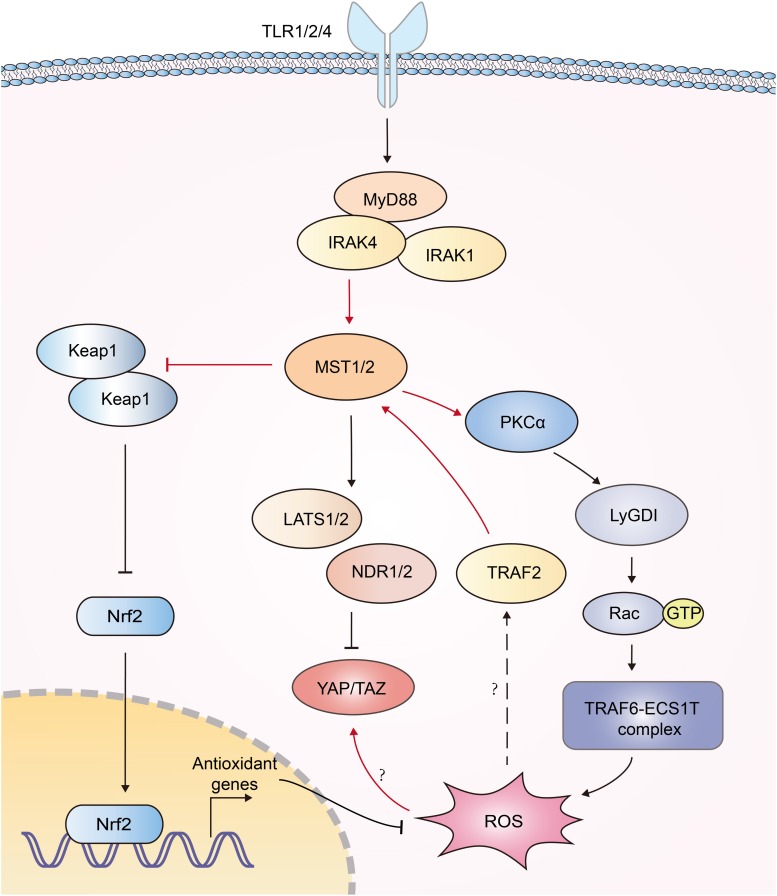
Interplays between Hippo signaling and ROS production. Recognition of bacterial infections by TLR1/2/4 triggers activation of MST1/2. Then MST1/2 phosphorylates PKC-α and activates PKCα-LyGDI-Rac cascade to induce assembly of the TRAF6-ECSIT complex, which is required for recruitment of mitochondria to phagosomes and ample ROS production. MST1 is activated by TRAF2 in response to H_2_O_2_ stimulation via unknown mechanism. MST1/2 phosphorylates Keap1 and prevents Keap1-mediated Nrf2 degradation, resulting in the expression of antioxidant genes. High intracellular ROS increase YAP expression independent of the canonical Hippo pathway; however, its mechanism is still elusive. The functions of Hippo-YAP in ROS production are highlighted with red color.

Reactive oxygen species (ROS) serves to eliminate invading pathogen in innate immunity (96). Mitochondria need to be juxtaposed to phagosomes for the synergistic production of ROS in phagocytes to kill pathogens (97, 98). Geng et al. documented that MST1/2 was vital for ROS-mediated innate anti-bacterial response (61). TLR1/2/4 sensed bacterial infections and triggered assembly of the TRAF6-ECSIT (evolutionarily conserved signaling intermediate in Toll pathways) complex, which was required for juxtaposition of mitochondria and phagosomes (97). MST1/2 was required for TLR-triggered assembly of the TRAF6-ECSIT complex by activating the GTPase Rac. After recognizing bacterial infection, TLRs activated MST1/2, which phosphorylated PKC-α and activated PKC-α-LyGDI (Rho-specific guanine nucleotide dissociation inhibitor)-Rac axis, promoting assembly of the TRAF6-ECSIT complex and ROS production (61) (Figure 5). Deletion of MST1 and MST2 diminished mitochondrion-phagosome juxtaposition and ROS induction. MST1- and MST2- deficient mice exhibited enhanced susceptibility to bacterial infection (61). Furthermore, MST1 was reported to be activated by ROS. H_2_O_2_ stimulation induced the binding of TRAF2 to MST1, which facilitated the dimerization and activation of MST1 (92). Recently, MST1/2 was demonstrated to sense ROS and protect macrophages from oxidative stress by modulating the stability of antioxidant transcription factor Nrf2 [nuclear factor (erythroid-derived 2)-like 2]. Phagosomal or mitochondrial ROS release activated MST1/2, which in turn phosphorylated Keap1 (kelch like ECH associated protein 1) and prevented Keap1-mediated Nrf2 from degradation. Nrf2 is the key transcriptional activator of the antioxidant response in macrophages. MST1/2-deficiency led to enhanced Nrf2 ubiquitination and low expression of antioxidant genes such as Nqo1, Ho-1, Gclc, and Gclm, resulting in increased oxidative stress, phagocyte aging and death (99). Interestingly, another group reported that high intracellular ROS increased YAP expression independent of the canonical Hippo pathway, implying more function of YAP independent of Hippo (100) (Figure 5). Generally, current studies implied a reciprocal activation of ROS and Hippo-YAP signaling (Figure 5).

## Perspectives of Hippo-YAP -Innate Immunity Crosstalk in Tumor Development

The transcription co-activators YAP and TAZ are found to be universally hyper-activated in various human malignancies. YAP/TAZ endows tumor cells with several crucial attributes, including survival, aberrant proliferation and stemness (101, 102). The upstream components of the Hippo-pathway including MST1/2, LATS1/2, NDR1/2, Merlin, and RASSFs have been identified to be tumor suppressors. Dysregulation of the Hippo pathway promote tumor initiation and progression (103–106), and is probably the major mechanism of resistance to various targeted and chemotherapies (107), making Hippo-YAP pathway an attractive anti-cancer target. Innate immunity plays vital roles in anti-tumor responses via detection of tumor DNA, induction of IFN-I, proinflammatory cytokines and chemokines, recruitment of inflammatory cells, and elicitation of adaptive immune responses (108).

As documented above, activation of many innate immune receptors activated Hippo-YAP pathway (55, 61, 70, 92), which might mediate the inactivation of YAP/TAZ and the suppression of tumor initiation and progression. Actually, the loss of function of innate immunity is closely related to tumor development. For instance, lacking of cGAS and/or STING has been found in more than a third of colorectal cancers (109). STING was reported to be frequently inactivated in HPV-induced cancers (110) and in a large portion of melanomas (111). Low expression of STING in hepatocellular carcinoma (HCC) tissues was closely related to more advanced tumor stages and a worse outcome (112). Low RIG-I expression led to shorter survival and poor prognosis of patients with HCC (113) and gastric cancer (114). RNA ligands-activated RIG-I and MDA5 could induce growth inhibition or apoptosis of multiple types of cancer cells in an IFN-independent manner (115, 116). In addition to these, tumor-derived DNA has been demonstrated to induce IFN-β production via cGAS-STING-IRF3 axis, contributing to spontaneous T cell- and natural killer cell mediated-anti-tumor responses. STING-deficient mice and IRF3-deficient mice exhibited defective tumor rejection (117–119). Several innate immune receptor ligands, such as poly (I:C), LPS and CpG, were proven to be efficient anti-tumor agents in different models of cancer [review in (120)]. Those ligands exhibited potential anti-tumor activity by inducing strong activation of IFN signaling and probably by activating the Hippo-YAP signaling. All these relations imply that loss of innate immunity in tumors might also lead to the activation of YAP or TAZ, therefore promote tumorigenesis. On the other hand, it is also reasonable that the accumulation of YAP/TAZ, usually caused by the dysregulation of Hippo-YAP pathway, might dampen the TBK1, IRF3 activation, and the anti-tumor responses. However, further investigation needs to be performed to demonstrate these hypotheses.

Although Hippo-YAP pathway has been convincingly proven to be a tumor suppressor signal, several studies reported that Hippo pathway attenuated anti-tumor immunity, and contribute to tumor initiation and progression in certain contexts, implying the complexity of anti-tumor immune regulation [review in (1, 121)]. Using three different murine syngeneic tumor models, Moroishi et al. found that LATS1/2 deletion or YAP/TAZ overexpression enhanced the immune response to tumor cells and inhibited tumor growth *in vivo*. Mechanically, LATS1/2-deficient tumor cells secreted robust level of nucleic-acid-rich extracellular vesicles, which activated IFN-I response via TLR-MYD88/TRIF signaling and enhanced antitumor immune responses (122). This study demonstrated a dual function of Hippo-YAP pathway in tumor development and immunosurveillance. Thus, in certain context, the potentiated immune response to tumor cells, induced by Hippo-YAP signaling deficiency, might overwhelm the tumor growth advantage and inhibit tumor growth.

On the contrary, several innate immune receptors were reported to correlate with tumor progression in multiple cancers (123). These might be attributed to the chronic inflammation induced by continuous activation of the receptors. More than 25% of cancers were associated with the persistent pathogen infection and chronic inflammation (124). For instance, persistent infection of hepatitis B virus (HBV) or hepatitis C (HCV) virus caused HCC (125, 126). Human papillomavirus (HPV) infection led to cervical cancer (127). These pathogens usually encode immune evasion mechanism and inhibit the antiviral or antibacterial signaling (110), which might further antagonize the Hippo-YAP signaling and promote tumor progression. Therefore, examining Hippo-YAP signaling status in virus-induced cancers will be necessary for further understanding this specific pathologic condition.

As documented above, innate immune responses act as a positive regulator of Hippo-YAP pathway in most cases. However, it could not explain all the issues, especially the TLRs which are correlated with either good or bad prognosis due to different types of cancer (128, 129). For example, high TLR9 expression indicated worse prognosis in oesophageal adenocarcinoma (130), but was associated with better survival in renal cell carcinoma (131). In lung cancer, TLR5 was correlated with good prognosis, but TLR7 with poor prognosis (132). Thus, our knowledge about the interplays of Hippo pathway and innate immunity may still in its infancy.

## Summary

The mutual regulation between Hippo-YAP pathway and innate immunity has been demonstrated by increasing amount of evidences. A number of clinical cases reported that lots of immunodeficiency symptoms, including recurrent bacterial and viral infections, were associated with the deficiency of Hippo signaling, implying its critical role in immune modulation (47, 48). Multiple proteins in Hippo-YAP signaling and innate immunity were identified to be involved in the crosstalk between Hippo-YAP pathway and innate immunity. In most cases, Hippo-YAP signaling potentiated the innate antiviral response, which could also positively feedback the Hippo-YAP pathway via multiple mechanisms. However, the interplay between Hippo-YAP and NF-κB signaling is intricate. Either signaling has been reported to positively or negatively regulate the other. The innate immunity probably influences tumor tumorigenesis, and progression via its interplay with Hippo signaling. The innate antiviral response leading to IRF3 activation and IFN production might indicate good outcome by provoking the Hippo signaling. However, the crosstalk between these two pathways still remains unclear and need further investigation. A comprehensive image of their regulations may pave the way for development of new cancer treatment.

## Author Contributions

SW, LZ, and FC wrote the manuscript. LL, XM, and SZ helped to revising the manuscript. FZ contributed to discussions and agreement with the conclusions.

## Conflict of Interest

The authors declare that the research was conducted in the absence of any commercial or financial relationships that could be construed as a potential conflict of interest.

## References

[1] Moroishi T. Hansen CG. Guan KL. The emerging roles of YAP and TAZ in cancer. *Nat Rev Cancer.* (2015) 15:73–9. 25592648 10.1038/nrc3876 PMC4562315

[2] Yu FX. Zhao B. Guan KL. Hippo pathway in organ size control, tissue homeostasis, and cancer. *Cell.* (2015) 163:811–28. 10.1016/j.cell.2015.10.044 26544935 PMC4638384

[3] Avruch J. Zhou D. Fitamant J. Bardeesy N. Mou F. Protein kinases of the Hippo pathway: regulation and substrates. *Semin Cell Dev Biol.* (2012) 23:770–84. 10.1016/j.semcdb.2012.07.002 22898666 PMC3489012

[4] Rauskolb C. Sun S. Sun G. Pan Y. Irvine KD. Cytoskeletal tension inhibits Hippo signaling through an Ajuba-Warts complex. *Cell.* (2014) 158:143–56. 10.1016/j.cell.2014.05.035 24995985 PMC4082802

[5] Wang L. Luo JY. Li B. Tian XY. Chen LJ. Integrin-YAP/TAZ-JNK cascade mediates atheroprotective effect of unidirectional shear flow. *Nature.* (2016) 540:579–82. 10.1038/nature20602 27926730

[6] Yu FX. Zhao B. Panupinthu N. Jewell JL. Lian I. Regulation of the Hippo-YAP pathway by G-protein-coupled receptor signaling. *Cell.* (2012) 150:780–91. 10.1016/j.cell.2012.06.037 22863277 PMC3433174

[7] Mo JS. Meng Z. Kim YC. Park HW. Hansen CG. Cellular energy stress induces AMPK-mediated regulation of YAP and the Hippo pathway. *Nat Cell Biol.* (2015) 17:500–10. 10.1038/ncb3111 25751140 PMC4380774

[8] Santinon G. Pocaterra A. Dupont S. Control of YAP/TAZ activity by metabolic and nutrient-sensing pathways. *Trends Cell Biol.* (2016) 26:289–99. 10.1016/j.tcb.2015.11.004 26750334

[9] Meng Z. Moroishi T. Mottier-Pavie V. Plouffe SW. Hansen CG. MAP4K family kinases act in parallel to MST1/2 to activate LATS1/2 in the Hippo pathway. *Nat Commun.* (2015) 6:8357. 10.1038/ncomms9357 26437443 PMC4600732

[10] Zheng Y. Wang W. Liu B. Deng H. Uster E. Identification of Happyhour/MAP4K as alternative Hpo/Mst-like kinases in the Hippo kinase cascade. *Dev Cell.* (2015) 34:642–55. 10.1016/j.devcel.2015.08.014 26364751 PMC4589524

[11] Hergovich A. Kohler RS. Schmitz D. Vichalkovski A. Cornils H. The MST1 and hMOB1 tumor suppressors control human centrosome duplication by regulating NDR kinase phosphorylation. *Curr Biol.* (2009) 19:1692–702. 10.1016/j.cub.2009.09.020 19836237

[12] Vichalkovski A. Gresko E. Cornils H. Hergovich A. Schmitz D. NDR kinase is activated by RASSF1A/MST1 in response to Fas receptor stimulation and promotes apoptosis. *Curr Biol.* (2008) 18:1889–95. 10.1016/j.cub.2008.10.060 19062280

[13] Hergovich A. Stegert MR. Schmitz D. Hemmings BA. NDR kinases regulate essential cell processes from yeast to humans. *Nat Rev Mol Cell Biol.* (2006) 7:253–64. 16607288 10.1038/nrm1891

[14] Zhang L. Tang F. Terracciano L. Hynx D. Kohler R. NDR functions as a physiological YAP1 kinase in the intestinal epithelium. *Curr Biol.* (2015) 25:296–305. 10.1016/j.cub.2014.11.054 25601544 PMC4426889

[15] Yin F. Yu J. Zheng Y. Chen Q. Zhang N. Spatial organization of Hippo signaling at the plasma membrane mediated by the tumor suppressor Merlin/NF2. *Cell.* (2013) 154:1342–55. 10.1016/j.cell.2013.08.025 24012335 PMC3835333

[16] Lallemand D. Curto M. Saotome I. Giovannini M. McClatchey AI. NF2 deficiency promotes tumorigenesis and metastasis by destabilizing adherens junctions. *Genes Dev.* (2003) 17:1090–100. 12695331 10.1101/gad.1054603 PMC196046

[17] Bitra A. Sistla S. Mariam J. Malvi H. Anand R. Rassf proteins as modulators of mst1 kinase activity. *Sci Rep.* (2017) 7:45020. 10.1038/srep45020 28327630 PMC5361201

[18] Brubaker SW. Bonham KS. Zanoni I. Kagan JC. Innate immune pattern recognition: a cell biological perspective. *Annu Rev Immunol.* (2015) 33:257–90. 10.1146/annurev-immunol-032414-112240 25581309 PMC5146691

[19] Akira S. Uematsu S. Takeuchi O. Pathogen recognition and innate immunity. *Cell.* (2006) 124:783–801. 10.1016/j.cell.2006.02.015 16497588

[20] Fischer S. Pattern recognition receptors and control of innate immunity: role of nucleic acids. *Curr Pharm Biotechnol.* (2018) 19:1203–209. 10.2174/138920112804583087 30636600

[21] Pandey S. Kawai T. Akira S. Microbial sensing by toll-like receptors and intracellular nucleic acid sensors. *Cold Spring Harb Perspect Biol.* (2014) 7:a016246. 10.1101/cshperspect.a016246 25301932 PMC4292165

[22] Alexopoulou L. Holt AC. Medzhitov R. Flavell RA. Recognition of double-stranded RNA and activation of NF-kappaB by Toll-like receptor 3. *Nature.* (2001) 413:732–38. 11607032 10.1038/35099560

[23] Heil F. Hemmi H. Hochrein H. Ampenberger F. Kirschning C. Species-specific recognition of single-stranded RNA via toll-like receptor 7 and 8. *Science.* (2004) 303:1526–529. 14976262 10.1126/science.1093620

[24] Diebold SS. Kaisho T. Hemmi H. Akira S. Reis e Sousa C. Innate antiviral responses by means of TLR7-mediated recognition of single-stranded RNA. *Science.* (2004) 303:1529–531. 14976261 10.1126/science.1093616

[25] Hemmi H. Takeuchi O. Kawai T. Kaisho T. Sato S. A Toll-like receptor recognizes bacterial DNA. *Nature.* (2000) 408:740–5. 11130078 10.1038/35047123

[26] Jin MS. Kim SE. Heo JY. Lee ME. Kim HM. Crystal structure of the TLR1-TLR2 heterodimer induced by binding of a tri-acylated lipopeptide. *Cell.* (2007) 130:1071–82. 17889651 10.1016/j.cell.2007.09.008

[27] Oliveira-Nascimento L. Massari P. Wetzler LM. The role of TLR2 in infection and immunity. *Front Immunol.* (2012) 3:79. 10.3389/fimmu.2012.00079 22566960 PMC3342043

[28] Poltorak A. He X. Smirnova I. Liu MY. Van Huffel C. Defective LPS signaling in C3H/HeJ and C57BL/10ScCr mice: mutations in Tlr4 gene. *Science.* (1998) 282:2085–88. 9851930 10.1126/science.282.5396.2085

[29] Hayashi F. Smith KD. Ozinsky A. Hawn TR. Yi EC. The innate immune response to bacterial flagellin is mediated by Toll-like receptor 5. *Nature.* (2001) 410:1099–103. 11323673 10.1038/35074106

[30] Kato H. Takeuchi O. Mikamo-Satoh E. Hirai R. Kawai T. Length-dependent recognition of double-stranded ribonucleic acids by retinoic acid-inducible gene-I and melanoma differentiation-associated gene 5. *J Exp Med.* (2008) 205:1601–10. 10.1084/jem.20080091 18591409 PMC2442638

[31] Wu J. Sun L. Chen X. Du F. Shi H. Cyclic GMP-AMP is an endogenous second messenger in innate immune signaling by cytosolic DNA. *Science.* (2013) 339:826–30. 10.1126/science.1229963 23258412 PMC3855410

[32] Sun L. Wu J. Du F. Chen X. Chen ZJ. Cyclic GMP-AMP synthase is a cytosolic DNA sensor that activates the type I interferon pathway. *Science.* (2013) 339:786–91. 10.1126/science.1232458 23258413 PMC3863629

[33] Unterholzner L. Keating SE. Baran M. Horan KA. Jensen SB. IFI16 is an innate immune sensor for intracellular DNA. *Nat Immunol.* (2010) 11:997–1004. 10.1038/ni.1932 20890285 PMC3142795

[34] Chiu YH. Macmillan JB. Chen ZJ. RNA polymerase III detects cytosolic DNA and induces type I interferons through the RIG-I pathway. *Cell.* (2009) 138:576–91. 10.1016/j.cell.2009.06.015 19631370 PMC2747301

[35] Takaoka A. Wang Z. Choi MK. Yanai H. Negishi H. DAI (DLM-1/ZBP1) is a cytosolic DNA sensor and an activator of innate immune response. *Nature.* (2007) 448:501–5. 17618271 10.1038/nature06013

[36] Zhang Z. Yuan B. Bao M. Lu N. Kim T. The helicase DDX41 senses intracellular DNA mediated by the adaptor STING in dendritic cells. *Nat Immunol.* (2011) 12:959–65. 10.1038/ni.2091 21892174 PMC3671854

[37] Seth RB. Sun L. Ea CK. Chen ZJ. Identification and characterization of MAVS, a mitochondrial antiviral signaling protein that activates NF-kappaB and IRF 3. *Cell.* (2005) 122:669–82. 16125763 10.1016/j.cell.2005.08.012

[38] Ishikawa H. Ma Z. Barber GN. STING regulates intracellular DNA-mediated, type I interferon-dependent innate immunity. *Nature.* (2009) 461:788–92. 10.1038/nature08476 19776740 PMC4664154

[39] Yamamoto M. Sato S. Hemmi H. Hoshino K. Kaisho T. Role of adaptor TRIF in the MyD88-independent toll-like receptor signaling pathway. *Science.* (2003) 301:640–43. 12855817 10.1126/science.1087262

[40] Medzhitov R. Preston-Hurlburt P. Kopp E. Stadlen A. Chen C. MyD88 is an adaptor protein in the hToll/IL-1 receptor family signaling pathways. *Mol Cell.* (1998) 2:253–58. 9734363 10.1016/s1097-2765(00)80136-7

[41] Fitzgerald KA. McWhirter SM. Faia KL. Rowe DC. Latz E. IKKepsilon and TBK1 are essential components of the IRF3 signaling pathway. *Nat Immunol.* (2003) 4:491–96. 12692549 10.1038/ni921

[42] Suzuki N. Suzuki S. Yeh WC. IRAK-4 as the central TIR signaling mediator in innate immunity. *Trends Immunol.* (2002) 23:503–6. 12297423 10.1016/s1471-4906(02)02298-6

[43] Zandi E. Rothwarf DM. Delhase M. Hayakawa M. Karin M. The IkappaB kinase complex (IKK) contains two kinase subunits, IKKalpha and IKKbeta, necessary for IkappaB phosphorylation and NF-kappaB activation. *Cell.* (1997) 91:243–52. 9346241 10.1016/s0092-8674(00)80406-7

[44] Kawai T. Akira S. Toll-like receptor and RIG-I-like receptor signaling. *Ann N Y Acad Sci.* (2008) 1143:1–20. 10.1196/annals.1443.020 19076341

[45] Ma Z. Damania B. The cGAS-STING defense pathway and its counteraction by viruses. *Cell Host Microbe.* (2016) 19:150–8. 10.1016/j.chom.2016.01.010 26867174 PMC4755325

[46] Di Cara F. Sheshachalam A. Braverman NE. Rachubinski RA. Simmonds AJ. Peroxisome-mediated metabolism is required for immune response to microbial infection. *Immunity.* (2017) 47:93.e7–106.e7. 10.1016/j.immuni.2017.06.016 28723556

[47] Abdollahpour H. Appaswamy G. Kotlarz D. Diestelhorst J. Beier R. The phenotype of human STK4 deficiency. *Blood.* (2012) 119:3450–57. 10.1182/blood-2011-09-378158 22294732 PMC3325036

[48] Nehme NT. Schmid JP. Debeurme F. Andre-Schmutz I. Lim A. MST1 mutations in autosomal recessive primary immunodeficiency characterized by defective naive T-cell survival. *Blood.* (2012) 119:3458–68. 10.1182/blood-2011-09-378364 22174160 PMC3824282

[49] Zhang Q. Meng F. Chen S. Plouffe SW. Wu S. Hippo signalling governs cytosolic nucleic acid sensing through YAP/TAZ-mediated TBK1 blockade. *Nat Cell Biol.* (2017) 19:362–74. 10.1038/ncb3496 28346439 PMC5398908

[50] Wang S. Xie F. Chu F. Zhang Z. Yang B. YAP antagonizes innate antiviral immunity and is targeted for lysosomal degradation through IKKvarepsilon-mediated phosphorylation. *Nat Immunol.* (2017) 18:733–43. 10.1038/ni.3744 28481329

[51] Fang C. Li J. Qi S. Lei Y. Zeng Y. An alternatively transcribed TAZ variant negatively regulates JAK-STAT signaling. *EMBO Rep.* (2019) 20:e47227. 10.15252/embr.201847227 30979708 PMC6549033

[52] Liu Z. Wu C. Pan Y. Liu H. Wang X. NDR2 promotes the antiviral immune response via facilitating TRIM25-mediated RIG-I activation in macrophages. *Sci Adv.* (2019) 5:eaav0163. 10.1126/sciadv.aav0163 30775439 PMC6365120

[53] Xu K. Xie X. Qi G. Weng P. Hu Z. Grass carp STK38 regulates IFN I expression by decreasing the phosphorylation level of GSK3beta. *Dev Comp Immunol.* (2019) 99:103410. 10.1016/j.dci.2019.103410 31175887

[54] Shalem O. Sanjana NE. Zhang F. High-throughput functional genomics using CRISPR-Cas9. *Nat Rev Genet.* (2015) 16:299–311. 10.1038/nrg3899 25854182 PMC4503232

[55] Li W. Xiao J. Zhou X. Xu M. Hu C. STK4 regulates TLR pathways and protects against chronic inflammation-related hepatocellular carcinoma. *J Clin Invest.* (2015) 125:4239–54. 10.1172/JCI81203 26457732 PMC4639976

[56] An H. Hou J. Zhou J. Zhao W. Xu H. Phosphatase SHP-1 promotes TLR- and RIG-I-activated production of type I interferon by inhibiting the kinase IRAK1. *Nat Immunol.* (2008) 9:542–50. 10.1038/ni.1604 18391954

[57] Bruni D. Sebastia J. Dunne S. Schroder M. Butler MP. A novel IRAK1-IKKepsilon signaling axis limits the activation of TAK1-IKKbeta downstream of TLR3. *J Immunol.* (2013) 190:2844–56. 10.4049/jimmunol.1202042 23396947

[58] Liu Y. Lu J. Zhang Z. Zhu L. Dong S. Amlexanox, a selective inhibitor of IKBKE, generates anti-tumoral effects by disrupting the Hippo pathway in human glioblastoma cell lines. *Cell Death Dis.* (2017) 8:e3022. 10.1038/cddis.2017.396 29048430 PMC5596579

[59] Yuan L. Mao Y. Luo W. Wu W. Xu H. Palmitic acid dysregulates the Hippo-YAP pathway and inhibits angiogenesis by inducing mitochondrial damage and activating the cytosolic DNA sensor cGAS-STING-IRF3 signaling mechanism. *J Biol Chem.* (2017) 292:15002–15. 10.1074/jbc.M117.804005 28698384 PMC5592676

[60] Jiao S. Guan J. Chen M. Wang W. Li C. Targeting IRF3 as a YAP agonist therapy against gastric cancer. *J Exp Med.* (2018) 215:699–718. 10.1084/jem.20171116 29339449 PMC5789414

[61] Geng J. Sun X. Wang P. Zhang S. Wang X. Kinases Mst1 and Mst2 positively regulate phagocytic induction of reactive oxygen species and bactericidal activity. *Nat Immunol.* (2015) 16:1142–52. 10.1038/ni.3268 26414765 PMC4618176

[62] Zhong J. Kyriakis JM. Germinal center kinase is required for optimal Jun N-terminal kinase activation by Toll-like receptor agonists and is regulated by the ubiquitin proteasome system and agonist-induced, TRAF6-dependent stabilization. *Mol Cell Biol.* (2004) 24:9165–75. 15456887 10.1128/MCB.24.20.9165-9175.2004 PMC517887

[63] Meng F. Zhou R. Wu S. Zhang Q. Jin Q. Mst1 shuts off cytosolic antiviral defense through IRF3 phosphorylation. *Genes Dev.* (2016) 30:1086–100. 10.1101/gad.277533.116 27125670 PMC4863739

[64] Boro M. Singh V. Balaji KN. Mycobacterium tuberculosis-triggered Hippo pathway orchestrates CXCL1/2 expression to modulate host immune responses. *Sci Rep.* (2016) 6:37695. 10.1038/srep37695 27883091 PMC5121601

[65] Hedengren M. Asling B. Dushay MS. Ando I. Ekengren S. Relish, a central factor in the control of humoral but not cellular immunity in *Drosophila*. *Mol Cell.* (1999) 4:827–37. 10619029 10.1016/s1097-2765(00)80392-5

[66] Valanne S. Wang JH. Ramet M. The *Drosophila* toll signaling pathway. *J Immunol.* (2011) 186:649–56. 10.4049/jimmunol.1002302 21209287

[67] Liu B. Zheng Y. Yin F. Yu J. Silverman N. Toll receptor-mediated hippo signaling controls innate immunity in *Drosophila*. *Cell.* (2016) 164:406–19. 10.1016/j.cell.2015.12.029 26824654 PMC4733248

[68] Dubey SK. Tapadia MG. Yorkie regulates neurodegeneration through canonical pathway and innate immune response. *Mol Neurobiol.* (2018) 55:1193–207. 10.1007/s12035-017-0388-7 28102471

[69] Ribeiro PS. Josue F. Wepf A. Wehr MC. Rinner O. Combined functional genomic and proteomic approaches identify a PP2A complex as a negative regulator of Hippo signaling. *Mol Cell.* (2010) 39:521–34. 10.1016/j.molcel.2010.08.002 20797625

[70] Lee IY. Lim JM. Cho H. Kim E. Kim Y. MST1 negatively regulates TNFalpha-induced NF-kappaB signaling through modulating LUBAC activity. *Mol Cell.* (2019) 73:1138.e6–49.e6. 10.1016/j.molcel.2019.01.022 30901564

[71] Hu MC. Wang Y. Qiu WR. Mikhail A. Meyer CF. Hematopoietic progenitor kinase-1 (HPK1) stress response signaling pathway activates IkappaB kinases (IKK-alpha/beta) and IKK-beta is a developmentally regulated protein kinase. *Oncogene.* (1999) 18:5514–24. 10523828 10.1038/sj.onc.1202740

[72] Arnold R. Liou J. Drexler HC. Weiss A. Kiefer F. Caspase-mediated cleavage of hematopoietic progenitor kinase 1 (HPK1) converts an activator of NFkappaB into an inhibitor of NFkappaB. *J Biol Chem.* (2001) 276:14675–84. 11278403 10.1074/jbc.M008343200

[73] Brenner D. Brechmann M. Rohling S. Tapernoux M. Mock T. Phosphorylation of CARMA1 by HPK1 is critical for NF-kappaB activation in T cells. *Proc Natl Acad Sci USA.* (2009) 106:14508–13. 10.1073/pnas.0900457106 19706536 PMC2732850

[74] Brenner D. Golks A. Kiefer F. Krammer PH. Arnold R. Activation or suppression of NFkappaB by HPK1 determines sensitivity to activation-induced cell death. *EMBO J.* (2005) 24:4279–90. 16341093 10.1038/sj.emboj.7600894 PMC1356335

[75] Chuang HC. Tan TH. MAP4K3/GLK in autoimmune disease, cancer and aging. *J Biomed Sci.* (2019) 26:82. 10.1186/s12929-019-0570-5 31640697 PMC6806545

[76] Chuang HC. Lan JL. Chen DY. Yang CY. Chen YM. The kinase GLK controls autoimmunity and NF-kappaB signaling by activating the kinase PKC-theta in T cells. *Nat Immunol.* (2011) 12:1113–8. 10.1038/ni.2121 21983831

[77] Wang X. Chuang HC. Li JP. Tan TH. Regulation of PKC-theta function by phosphorylation in T cell receptor signaling. *Front Immunol.* (2012) 3:197. 10.3389/fimmu.2012.00197 22798961 PMC3393885

[78] Chen YM. Chuang HC. Lin WC. Tsai CY. Wu CW. Germinal center kinase-like kinase overexpression in T cells as a novel biomarker in rheumatoid arthritis. *Arthritis Rheum.* (2013) 65:2573–82. 10.1002/art.38067 23817999

[79] Chuang HC. Tsai CY. Hsueh CH. Tan TH. GLK-IKKbeta signaling induces dimerization and translocation of the AhR-RORgammat complex in IL-17A induction and autoimmune disease. *Sci Adv.* (2018) 4:eaat5401. 10.1126/sciadv.aat5401 30214937 PMC6135549

[80] Aouadi M. Tesz GJ. Nicoloro SM. Wang M. Chouinard M. Orally delivered siRNA targeting macrophage Map4k4 suppresses systemic inflammation. *Nature.* (2009) 458:1180–4. 10.1038/nature07774 19407801 PMC2879154

[81] Ma C. Lin W. Liu Z. Tang W. Gautam R. NDR1 protein kinase promotes IL-17- and TNF-alpha-mediated inflammation by competitively binding TRAF3. *EMBO Rep.* (2017) 18:586–602. 10.15252/embr.201642140 28219902 PMC5376972

[82] Zhu S. Pan W. Shi P. Gao H. Zhao F. Modulation of experimental autoimmune encephalomyelitis through TRAF3-mediated suppression of interleukin 17 receptor signaling. *J Exp Med.* (2010) 207:2647–62. 10.1084/jem.20100703 21078888 PMC2989772

[83] Wen M. Ma X. Cheng H. Jiang W. Xu X. Stk38 protein kinase preferentially inhibits TLR9-activated inflammatory responses by promoting MEKK2 ubiquitination in macrophages. *Nat Commun.* (2015) 6:7167. 10.1038/ncomms8167 25981615

[84] Schmidt C. Peng B. Li Z. Sclabas GM. Fujioka S. Mechanisms of proinflammatory cytokine-induced biphasic NF-kappaB activation. *Mol Cell.* (2003) 12:1287–300. 14636585 10.1016/s1097-2765(03)00390-3

[85] Ma X. Wang D. Li N. Gao P. Zhang M. Hippo kinase NDR2 inhibits IL-17 signaling by promoting Smurf1-mediated MEKK2 ubiquitination and degradation. *Mol Immunol.* (2019) 105:131–6. 10.1016/j.molimm.2018.10.005 30504095

[86] Lv Y. Kim K. Sheng Y. Cho J. Qian Z. YAP Controls endothelial activation and vascular inflammation through TRAF6. *Circ Res.* (2018) 123:43–56. 10.1161/CIRCRESAHA.118.313143 29794022 PMC6014930

[87] Deng Y. Lu J. Li W. Wu A. Zhang X. Reciprocal inhibition of YAP/TAZ and NF-kappaB regulates osteoarthritic cartilage degradation. *Nat Commun.* (2018) 9:4564. 10.1038/s41467-018-07022-2 30385786 PMC6212432

[88] Kapoor M. Martel-Pelletier J. Lajeunesse D. Pelletier JP. Fahmi H. Role of proinflammatory cytokines in the pathophysiology of osteoarthritis. *Nat Rev Rheumatol.* (2011) 7:33–42. 10.1038/nrrheum.2010.196 21119608

[89] Zhang Q. Han X. Chen J. Xie X. Xu J. Yes-associated protein (YAP) and transcriptional coactivator with PDZ-binding motif (TAZ) mediate cell density-dependent proinflammatory responses. *J Biol Chem.* (2018) 293:18071–85. 10.1074/jbc.RA118.004251 30315101 PMC6254335

[90] Hagenbeek TJ. Webster JD. Kljavin NM. Chang MT. Pham T. The Hippo pathway effector TAZ induces TEAD-dependent liver inflammation and tumors. *Sci Signal.* (2018) 11:eaaj1757. 10.1126/scisignal.aaj1757 30206136

[91] Mooring M. Fowl BH. Lum SZC. Liu Y. Yao K. Hepatocyte stress increases expression of yap and taz in hepatocytes to promote parenchymal inflammation and fibrosis. *Hepatology.* (2019). 10.1002/hep.30928 31505040 PMC7062580

[92] Roh KH. Choi EJ. TRAF2 functions as an activator switch in the reactive oxygen species-induced stimulation of MST1. *Free Radic Biol Med.* (2016) 91:105–13. 10.1016/j.freeradbiomed.2015.12.010 26698664

[93] Diener K. Wang XS. Chen C. Meyer CF. Keesler G. Activation of the c-Jun N-terminal kinase pathway by a novel protein kinase related to human germinal center kinase. *Proc Natl Acad Sci USA.* (1997) 94:9687–92. 9275185 10.1073/pnas.94.18.9687 PMC23251

[94] Shi CS. Leonardi A. Kyriakis J. Siebenlist U. Kehrl JH. TNF-mediated activation of the stress-activated protein kinase pathway: TNF receptor-associated factor 2 recruits and activates germinal center kinase related. *J Immunol.* (1999) 163:3279–85. 10477597

[95] Shi CS. Kehrl JH. Tumor necrosis factor (TNF)-induced germinal center kinase-related (GCKR) and stress-activated protein kinase (SAPK) activation depends upon the E2/E3 complex Ubc13-Uev1A/TNF receptor-associated factor 2 (TRAF2). *J Biol Chem.* (2003) 278:15429–34. 12591926 10.1074/jbc.M211796200

[96] Yang Y. Bazhin AV. Werner J. Karakhanova S. Reactive oxygen species in the immune system. *Int Rev Immunol.* (2013) 32:249–70. 10.3109/08830185.2012.755176 23617726

[97] West AP. Brodsky IE. Rahner C. Woo DK. Erdjument-Bromage H. TLR signalling augments macrophage bactericidal activity through mitochondrial ROS. *Nature.* (2011) 472:476–80. 10.1038/nature09973 21525932 PMC3460538

[98] Sena LA. Chandel NS. Physiological roles of mitochondrial reactive oxygen species. *Mol Cell.* (2012) 48:158–67. 10.1016/j.molcel.2012.09.025 23102266 PMC3484374

[99] Wang P. Geng J. Gao J. Zhao H. Li J. Macrophage achieves self-protection against oxidative stress-induced ageing through the Mst-Nrf2 axis. *Nat Commun.* (2019) 10:755. 10.1038/s41467-019-08680-6 PMC6376064 30765703

[100] Dixit D. Ghildiyal R. Anto NP. Sen E. Chaetocin-induced ROS-mediated apoptosis involves ATM-YAP1 axis and JNK-dependent inhibition of glucose metabolism. *Cell Death Dis.* (2014) 5:e1212 10.1038/cddis.2014.179 PMC4047915 24810048

[101] Zanconato F. Cordenonsi M. Piccolo S. YAP/TAZ at the roots of cancer. *Cancer Cell.* (2016) 29:783–803. 10.1016/j.ccell.2016.05.005 27300434 PMC6186419

[102] Hanahan D. Weinberg RA. Hallmarks of cancer: the next generation. *Cell.* (2011) 144:646–764. 21376230 10.1016/j.cell.2011.02.013

[103] O’Neill EE. Matallanas D. Kolch W. Mammalian sterile 20-like kinases in tumor suppression: an emerging pathway. *Cancer Res.* (2005) 65:5485–7. 15994916 10.1158/0008-5472.CAN-05-1453

[104] Hergovich A. Hemmings BA. Mammalian NDR/LATS protein kinases in hippo tumor suppressor signaling. *Biofactors.* (2009) 35:338–45. 10.1002/biof.47 19484742

[105] Harvey KF. Zhang X. Thomas DM. The Hippo pathway and human cancer. *Nat Rev Cancer.* (2013) 13:246–57. 23467301 10.1038/nrc3458

[106] Iwasa H. Hossain S. Hata Y. Tumor suppressor C-RASSF proteins. *Cell Mol Life Sci.* (2018) 75:1773–87. 10.1007/s00018-018-2756-5 29353317 PMC11105443

[107] Nguyen CDK. Yi C. YAP/TAZ signaling and resistance to cancer therapy. *Trends Cancer.* (2019) 5:283–96. 31174841 10.1016/j.trecan.2019.02.010 PMC6557283

[108] Demaria O. Cornen S. Daeron M. Morel Y. Medzhitov R. Harnessing innate immunity in cancer therapy. *Nature.* (2019) 574:45–56. 10.1038/s41586-019-1593-5 31578484

[109] Xia T. Konno H. Ahn J. Barber GN. Deregulation of STING signaling in colorectal carcinoma constrains DNA damage responses and correlates with tumorigenesis. *Cell Rep.* (2016) 14:282–97. 10.1016/j.celrep.2015.12.029 26748708 PMC4845097

[110] Lau L. Gray EE. Brunette RL. Stetson DB. DNA tumor virus oncogenes antagonize the cGAS-STING DNA-sensing pathway. *Science.* (2015) 350:568–71. 10.1126/science.aab3291 26405230 PMC12974531

[111] Xia T. Konno H. Barber GN. Recurrent loss of STING signaling in melanoma correlates with susceptibility to viral oncolysis. *Cancer Res.* (2016) 76:6747–59. 10.1158/0008-5472.CAN-16-1404 27680683

[112] Bu Y. Liu F. Jia QA. Yu SN. Decreased expression of TMEM173 predicts poor prognosis in patients with hepatocellular carcinoma. *PLoS One.* (2016) 11:e0165681. 10.1371/journal.pone.0165681 27814372 PMC5096716

[113] Hou J. Zhou Y. Zheng Y. Fan J. Zhou W. Hepatic RIG-I predicts survival and interferon-alpha therapeutic response in hepatocellular carcinoma. *Cancer Cell.* (2014) 25:49–63. 10.1016/j.ccr.2013.11.011 24360797

[114] Chen L. Feng J. Wu S. Xu B. Zhou Y. Decreased RIG-I expression is associated with poor prognosis and promotes cell invasion in human gastric cancer. *Cancer Cell Int.* (2018) 18:144. 10.1186/s12935-018-0639-3 30250402 PMC6146491

[115] Wu Y. Wu X. Wu L. Wang X. Liu Z. The anticancer functions of RIG-I-like receptors, RIG-I and MDA5, and their applications in cancer therapy. *Transl Res.* (2017) 190:51–60. 10.1016/j.trsl.2017.08.004 28917654

[116] Besch R. Poeck H. Hohenauer T. Senft D. Hacker G. Proapoptotic signaling induced by RIG-I and MDA-5 results in type I interferon-independent apoptosis in human melanoma cells. *J Clin Invest.* (2009) 119:2399–411. 10.1172/JCI37155 19620789 PMC2719920

[117] Woo SR. Fuertes MB. Corrales L. Spranger S. Furdyna MJ. STING-dependent cytosolic DNA sensing mediates innate immune recognition of immunogenic tumors. *Immunity.* (2014) 41:830–42. 10.1016/j.immuni.2014.10.017 25517615 PMC4384884

[118] Marcus A. Mao AJ. Lensink-Vasan M. Wang L. Vance RE. Tumor-derived cGAMP triggers a STING-mediated interferon response in non-tumor cells to activate the NK cell response. *Immunity.* (2018) 49:754.e4–63.e4. 10.1016/j.immuni.2018.09.016 30332631 PMC6488306

[119] Li A. Yi M. Qin S. Song Y. Chu Q. Activating cGAS-STING pathway for the optimal effect of cancer immunotherapy. *J Hematol Oncol.* (2019) 12:35. 10.1186/s13045-019-0721-x 30935414 PMC6444510

[120] Urban-Wojciuk Z. Khan MM. Oyler BL. Fahraeus R. Marek-Trzonkowska N. The role of TLRs in anti-cancer immunity and tumor rejection. *Front Immunol.* (2019) 10:2388. 10.3389/fimmu.2019.02388 31695691 PMC6817561

[121] Wang H. Du YC. Zhou XJ. Liu H. Tang SC. The dual functions of YAP-1 to promote and inhibit cell growth in human malignancy. *Cancer Metastasis Rev.* (2014) 33:173–81. 10.1007/s10555-013-9463-3 24346160

[122] Moroishi T. Hayashi T. Pan WW. Fujita Y. Holt MV. The Hippo pathway kinases LATS1/2 suppress cancer immunity. *Cell.* (2016) 167:1525.e7–39.e7. 27912060 10.1016/j.cell.2016.11.005 PMC5512418

[123] Li TT. Ogino S. Qian ZR. Toll-like receptor signaling in colorectal cancer: carcinogenesis to cancer therapy. *World J Gastroenterol.* (2014) 20:17699–708. 10.3748/wjg.v20.i47.17699 25548469 PMC4273121

[124] Hussain SP. Harris CC. Inflammation and cancer: an ancient link with novel potentials. *Int J Cancer.* (2007) 121:2373–80. 17893866 10.1002/ijc.23173

[125] Chisari FV. Rous-whipple award lecture. Viruses, immunity, and cancer: lessons from hepatitis B. *Am J Pathol.* (2000) 156:1117–32. 10751335 10.1016/s0002-9440(10)64980-2 PMC1876872

[126] Castello G. Scala S. Palmieri G. Curley SA. Izzo F. HCV-related hepatocellular carcinoma: from chronic inflammation to cancer. *Clin Immunol.* (2010) 134:237–50. 19910258 10.1016/j.clim.2009.10.007

[127] Castellsague X. Natural history and epidemiology of HPV infection and cervical cancer. *Gynecol Oncol.* (2008) 110(Suppl. 2):S4–7. 10.1016/j.ygyno.2008.07.045 18760711

[128] Rakoff-Nahoum S. Medzhitov R. Toll-like receptors and cancer. *Nat Rev Cancer.* (2009) 9:57–63. 19052556 10.1038/nrc2541

[129] Pradere JP. Dapito DH. Schwabe RF. The yin and yang of toll-like receptors in cancer. *Oncogene.* (2014) 33:3485–95. 10.1038/onc.2013.302 23934186 PMC4059777

[130] Kauppila JH. Takala H. Selander KS. Lehenkari PP. Saarnio J. Increased Toll-like receptor 9 expression indicates adverse prognosis in oesophageal adenocarcinoma. *Histopathology.* (2011) 59:643–9. 10.1111/j.1365-2559.2011.03991.x 22014045

[131] Ronkainen H. Hirvikoski P. Kauppila S. Vuopala KS. Paavonen TK. Absent Toll-like receptor-9 expression predicts poor prognosis in renal cell carcinoma. *J Exp Clin Cancer Res.* (2011) 30:84. 10.1186/1756-9966-30-84 21929816 PMC3182949

[132] Gu J. Liu Y. Xie B. Ye P. Huang J. Roles of toll-like receptors: from inflammation to lung cancer progression. *Biomed Rep.* (2018) 8:126–32. 10.3892/br.2017.1034 29435270 PMC5778860

